# “Project YES! has given me a task to reach undetectable”: Qualitative findings from a peer mentoring program for youth living with HIV in Zambia

**DOI:** 10.1371/journal.pone.0292719

**Published:** 2023-10-13

**Authors:** Katherine G. Merrill, Christiana Frimpong, Virginia M. Burke, Elizabeth A. Abrams, Sam Miti, Jonathan K. Mwansa, Julie A. Denison

**Affiliations:** 1 Department of International Health, Johns Hopkins Bloomberg School of Public Health, Baltimore, Maryland, United States of America; 2 Arthur Davison Children’s Hospital, Ndola, Zambia; University of Sharjah, UNITED ARAB EMIRATES

## Abstract

The Project YES! clinic-based peer mentoring program was a randomized controlled trial (RCT) conducted among 276 youth from four HIV clinics to test the impact of the program on promoting HIV self-management and reducing internalized stigma among youth living with HIV (ages 15–24 years) in Ndola, Zambia. We conducted a qualitative sub-study involving in-depth interviews with 40 intervention youth participants (21 female, 19 male) to explore their experiences with Project YES! which included: an orientation meeting led by a healthcare provider, monthly individual and group counseling sessions over six months, and three optional caregiver group sessions. Using baseline RCT data, we used maximum variation sampling to purposively select youth by sex, age, change in virologic results between baseline and midline, and study clinic. A four-person team conducted thematic coding. Youth described their increased motivation to take their HIV care seriously due to Project YES!, citing examples of improvements in ART adherence and for some, virologic results. Many cited changes in behavior in the context of greater feelings of self-worth and acceptance of their HIV status, resulting in less shame and fear associated with living with HIV. Youth also attributed Project YES! with reducing their sense of isolation and described Project YES! youth peer mentors and peers as their community and “family.” Findings highlight that self-worth and personal connections play a critical role in improving youths’ HIV outcomes. Peer-led programs can help foster these gains through a combination of individual and group counseling sessions. Greater attention to the context in which youth manage their HIV, beyond medication intake, is needed to reach global HIV targets.

## Introduction

While progress has been made in the past decade in reducing the number of new HIV infections among young people (aged 15–24 years), this age group still accounted for two out of every seven new HIV infections in 2019 [1]. Compared to other age groups, youth living with HIV in sub-Saharan Africa, home to 88% of youth living with HIV worldwide [2], experience more incomplete antiretroviral therapy (ART) treatment and virologic failure [3, 4]. All adolescents and young adults are in a unique period of tremendous emotional, social, cognitive, and psychological change [5, 6], but those living with HIV face added pressures of coming to terms with their HIV status and building their sense of identity [7], which can exacerbate the barriers they face with their HIV care and treatment. Indeed, numerous factors have been associated with incomplete ART adherence and/or virologic failure among youth in the region, including stigma, treatment fatigue, non-disclosure to family members, and a lack of caregiver assistance or people to talk with about living with HIV after disclosure [8–10].

Despite increasing recognition of the need to focus on the HIV epidemic among young people to reach global HIV targets [11, 12], systematic reviews have found few high-quality intervention studies which address youths’ HIV care and treatment outcomes [13–15]. To help fill this gap, our team developed and tested through a randomized controlled trial (RCT) Project YES! Youth Engaging for Success for adolescents and young adults (AYA) living with HIV in Zambia (NCRT #04115813; Clinicaltrials.gov identifier: NCT04115813) [16]. Zambia has among the highest HIV prevalence rates globally (11.1% among people 15–59 years) [17], and in 2016 only about one-third of Zambian youth aged 15–24 years had achieved viral suppression compared to nearly three-quarters of adults [18]. Project YES! trained and paid young adults living with HIV to work in clinics as peer mentors to facilitate youths’ HIV self-management [19]. While evidence for the use of peer interventions among people living with HIV broadly is mixed [20], studies have shown promising findings among youth living with HIV in sub-Saharan Africa [21, 22].

The Project YES! RCT found that the relative odds of experiencing feelings of internalized stigma—measured via three items: 1) you feel guilty that you are HIV positive; 2) you feel ashamed that you are HIV positive; and 3) you sometimes feel worthless because you are HIV positive—were significantly lower among Project YES! intervention versus comparison group participants (OR: 0.39, 95% CI: 0.21–0.73). Moreover, a sub-group of Project YES! participants in the pediatric setting had a relative increase of 4.7 in the odds of viral suppression (95% CI: 1.84–11.78) compared to comparison group participants [16]. This study reports on qualitative data collected from youth participants in the Project YES! RCT. We explored youths’ experiences with Project YES! to strengthen our understanding of the intervention’s effectiveness and implementation, while enhancing the literature on peer-centered approaches to improving HIV outcomes among youth living with HIV.

## Materials and methods

### Overview of Project YES!

**Fig 1:**
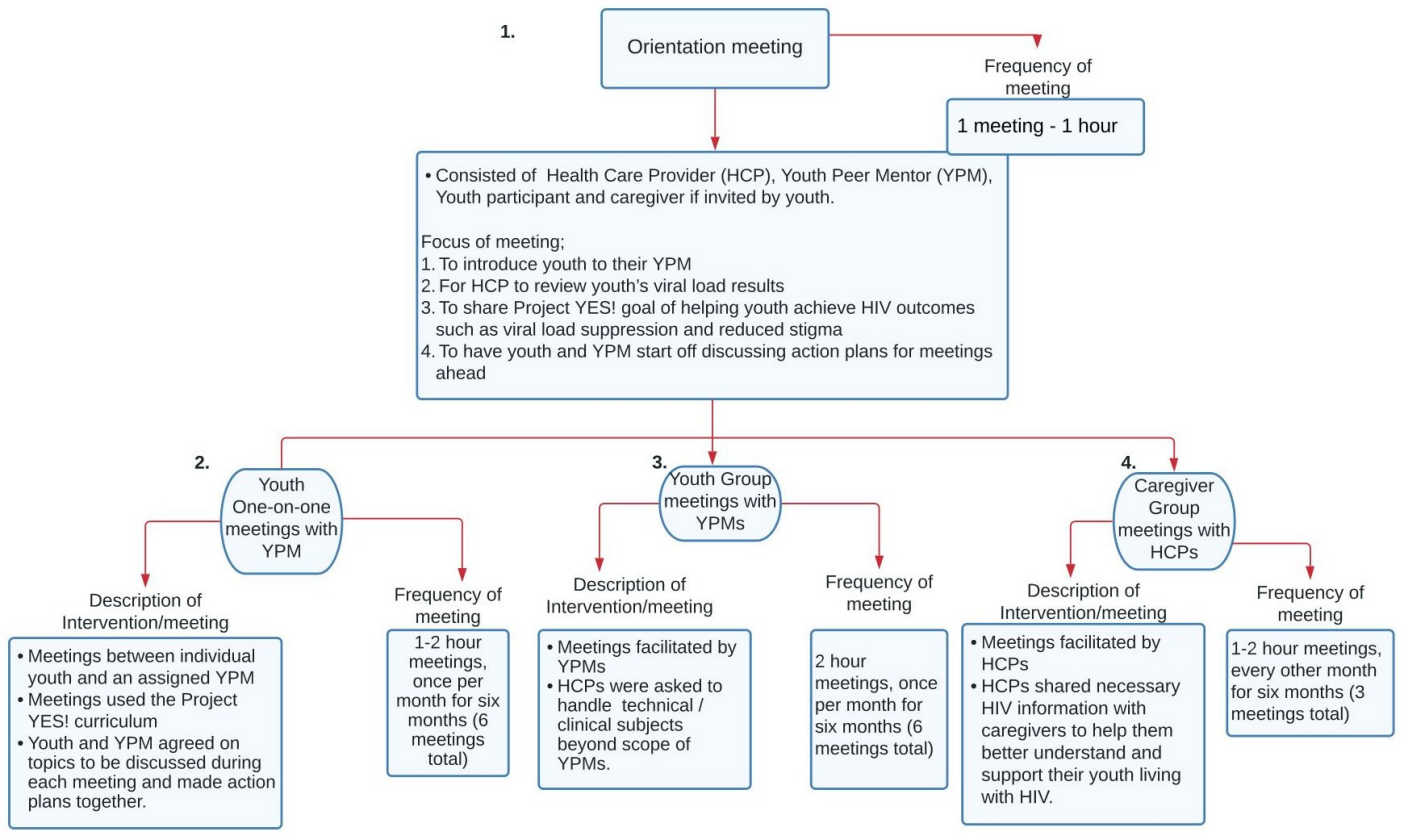
Overview of Project YES! intervention. The intervention was delivered to the study intervention arm for the first six months and to the comparison arm for the following six months while the intervention arm completed a maintenance period.

The Project YES! RCT consecutively enrolled 276 youth living with HIV from four HIV clinics—a children’s hospital, an adult hospital, and two primary health facilities—in Ndola, Zambia (full study details elsewhere [23]). Youth were eligible if aged 15–24 years, an English or Bemba speaker, aware of their HIV status, on ART for 6+ months, and available for the 18-month study. Participants randomized to the intervention arm received a six-month peer-mentoring program, which included an orientation meeting (with optional caregiver participation), and monthly individual and monthly group meetings with a youth peer mentor (YPM). Caregivers, if invited by the youth, could attend three caregiver group meetings (Fig 1). The intervention draws on the Five Cs of positive youth development, including competence, confidence, connection, character, and caring [24]. It also draws on elements of Social Cognitive Theory, including agency to perform a behavior through essential knowledge and skills, observational learning through modeling of behaviors by peer mentors, and self-efficacy to perform a behavior through increased confidence [25, 26].

YPM, aged 21–26 years, who were identified by healthcare providers (HCP) in the four study clinics as successfully managing their HIV, completed a two-week training and were hired by Project YES! to work in the clinics. They underwent one month of practice meetings with youth before the intervention’s launch. HCP led the orientation meeting and caregiver meetings and were available to answer youths’ questions if outside the scope of YPM knowledge. Prior to the intervention, youth attending the children’s hospital were assessed for a possible physical transition to an adult hospital. Those with viral load failure at baseline were assessed for drug resistance and recommended an ART medication change if needed [27]. Assessments, including surveys and blood draws for virologic testing, were conducted at baseline, ~6 months, and ~12 months. While the intervention arm received the intervention, the comparison arm received usual care. After six months, the intervention arm entered a six-month maintenance period consisting of individual or group meetings every other month, and the comparison arm received the intervention. After another six months, once the comparison arm had received the intervention, Project YES! activities ended, and participants completed the endline assessment. Alongside the RCT, qualitative data were collected from youth participants, in addition to YPM, HCP, and caregivers of youth participants (findings from YPM and HCP are presented elsewhere [19, 28]), to explore experiences with the Project YES! intervention.

### Qualitative data collection and analysis

Forty youth (21 female, 19 male) randomized to the Project YES! intervention arm participated in one-time semi-structured in-depth interviews (IDI) about their experiences with the program. Interviews were chosen for data collection given their usefulness for exploring sensitive topics (e.g., HIV stigma, ART adherence, etc.), where participants may not wish to discuss such issues in a group environment [29]. Maximum variation sampling was used to achieve heterogeneity in views about the program. Accordingly, youth were purposively selected to ensure balanced representation from across the following characteristics, determined from their reports on the baseline questionnaire: sex, age group (15–19 or 20–24 years), change in virologic results between baseline and midline (~six months post), and study clinic. Three Zambian interviewers who had previous research experience with young people and were unaffiliated with the Project YES! programs team conducted the IDI following five days of study-specific training. Interviewers were matched with participants by sex. IDI were conducted in English or Bemba (based on youth preference) in a private space at the clinics. They lasted 30–75 minutes and were audio-recorded with youth’s permission and transcribed in English by a Lusaka-based transcription company. Transcripts were reviewed by the interviewers to confirm their accuracy. IDI were held from January to March 2019, after the completion of the six-month intervention and midline RCT data collection during the intervention arm’s maintenance period.

The IDI guide asked about youth’s experiences with the program generally and with the individual and group meetings, specifically. It probed into any changes resulting from the program, perspectives of caregiver involvement, experiences with HIV self-management, HCP interactions, and program recommendations (sample interview questions are in Table 1). Two topics in the guide—experiences with a drug change (if applicable) and with the study’s safety protocol [30]—are the focus of other analyses.

**Table 1:** Sample interview questions for youth participants by domain of interest.

Domain	Sample interview question
Overall experiences in program	Tell me about your experience in the Project YES! peer mentoring program. What did you like and dislike about the program?
One-on-one and group meetings	What do you prefer: the one-on-one sessions, the group sessions, or both? Please explain.
How things changed or stayed the same	How has the project affected your health? What changed or stayed the same about how you care for your health and HIV after talking with your peer mentor?
Caregiver involvement	Tell me about a time you discussed Project YES! with your caregiver. How did your caregiver feel about your participation in Project YES!?
HIV self-management	Tell me about the last time you missed taking your HIV medication. What happened?
Healthcare provider interactions	How do you feel about your interactions with health care providers at your clinic? How comfortable do you feel asking questions during your visit if you want something to be clearer?
Program recommendations	Would you want this program to continue or stop? Why or why not?

Transcripts were thematically coded by a trained four-person team. We used deductive and inductive approaches by creating a codebook based on the IDI guide and adding codes iteratively [31]. We drew on Tobin and Begley’s criteria for ensuring qualitative rigor [32]. The coding team and principal investigator (JD) met regularly to develop a common understanding of the codes and their application and to discuss emerging patterns. These meetings were used to achieve *credibility*—i.e., ensuring that the researchers’ representation of the themes fit with the respondents’ views expressed during the interviews—and *confirmability*—i.e., that the interpretations were clearly derived from the data [32]. Memoing was used to consolidate emerging themes and as a reflexivity tool, whereby the researchers kept a “self-critical” account of the research process and reflected on potential biases vis-à-vis the research topic to enhance the *dependability* of findings [32]. Findings were compared with those derived from in-depth interviews with YPM and HCP to achieve *completeness* in recognizing the full complexity of an exploration of experiences in the Project YES! intervention [32]. NVivo 14 facilitated the coding and organization of findings.

### Ethics

Participants provided written informed consent to enroll in the RCT, including to participate in an IDI. Written parental consent and youth assent were obtained for 15-17-year olds [33]. At the beginning of the IDI, interviewers read a script to remind participants of key ethical issues and asked participants to verbally agree to proceed. Interviewers were trained to bring IDI participants who described severe experiences of violence (e.g., forced sex, physical beating) or suicidal ideation to HCP at the clinic according to the study’s safety protocol. The providers handled these cases according to clinical practice, local policy, and Zambian law, and provided onward referrals as needed [30]. Participants were reimbursed 50 Kwacha (roughly $5.00 USD) for their time and transportation to the clinic. This research was approved by the ethics review boards at ERES Converge in Zambia (2017-Sept-012) and the Johns Hopkins Bloomberg School of Public Health in the United States (00007870), and the Zambia Ministry of Health through the National Health Research Authority.

## Results

The predominant themes emerging across the IDI concerned youths’: a) increased motivation for HIV care, b) reduced feelings of shame and fear and enhanced self-worth, and c) greater sense of community, which they attributed to the Project YES! intervention. Respondent characteristics are in Table 2. Findings did not differ based on the youths’ sex, age, change in viral load status during the project, or study clinic.

**Table 2:** Interview respondent characteristics according to sampling criteria.

Sampling characteristic	n (%)
Sex	
Female	21 (53%)
Male	19 (47%)
Age group	
15–19 years	24 (60%)
20–24 years	16 (40%)
Change in viral load from baseline to midline (~6 months later)	
Failure to suppression	10 (25%)
Suppression to suppression	16 (40%)
Suppression to failure	7 (18%)
Failure to failure	6 (15%)
Missing	1 (3%)
Study clinic	
Child hospital	14 (35%)
Adult clinic	15 (38%)
Transitioned*	11 (27%)

*From children’s hospital to adult hospital.

### “A task to reach undetectable”: Gaining the motivation to take their HIV care seriously

As the most salient theme, youth described how Project YES! had inspired them to be mindful of their HIV care. Most described taking their medication inconsistently before the project—they were “not paying attention” and “didn’t really care”—but many described improvements through the project. As a 16-year-old male participant explained, “I am concerned about my health…When I get home, I no longer forget [to take my medication]. I manage everything.” Among the subset sampled whose viral load went from failure to suppression, most gave examples of how their viral load had gone down since participating in Project YES!, which had encouraged them to remain diligent with their ART. “Project YES! has…given me a task to reach undetectable,” said a 21-year-old male participant. A few youth explained that their family members no longer needed to “force” them to take their medication since they now had the self-motivation to carry through with the task.

The youth also described increased motivation to take care of themselves in other ways. Many, for instance, described learning about nutrition as it relates to their health. They learned about the importance of eating a balanced diet and consuming sufficient food to support ART adherence—i.e., “foods which can help us to have energy so that this medicine can work in our bodies” (female, 21 years). A 23-year-old explained how she used to feel “that diet was not necessary,” but “from the time I started attending this [program], I am able to know…if I don’t eat right…my health will be affected. And since then, my health has been better.”

Youth attributed their increased motivation for self-care to a combination of factors. For most, the knowledge gained from the program enhanced their motivation for self-care. They learned, for instance, the importance of taking their ART medication “properly” and “on time” because “we know the effects of not doing so.” Many gained practical skills to apply in their daily lives. They cited strategies for taking their medication (e.g., setting an alarm, keeping a record/diary) and managing their stress. A 19-year-old male participant, for example, described the importance of factoring his medication intake into his daily schedule; if he knew he is due for his medication at 20 hours but was leaving home at 19 hours and was unsure of his plans, he should bring the medication with him.

Youth also highlighted the supportive role of the YPM and HCP and their recognition of the difficulties that youth face; as a 17-year-old female participant explained, “They tell us…every person has a challenge…. I’m grateful because I did not know that they would be this encouraging to me.” In particular, the youth described the benefits of meeting with a YPM who was also living with HIV and only slightly older than them. It made them feel “comfortable” sharing information about their lives and the challenges they face, knowing that the YPM would “understand more” than someone who was not living with HIV. According to a 21-year-old male participant, “If you are opening the secret to someone who is in the same situation as you, it just comes out easily, with no hesitation, fear, or embarrassment.” Finally, the youth developed future aspirations. They learned that every person has a right to marry, no matter their HIV status, and that it is possible to avoid giving HIV to a partner and to a baby. This knowledge allowed them to envision healthy future lives with their HIV status.

### “I have learned not to fear anyone or anything”: Overcoming shame and fears of living with HIV, and developing greater feelings of self-worth

The second most-salient theme, which builds on the first, centered on how Project YES! helped many youth accept their HIV status and “stop being scared.” The youth learned to identify self-stigma and accept their HIV status “from the heart,” while gaining self-confidence and greater feelings of self-worth. They learned to “ignore” negative comments from people about HIV, and no longer feared socializing with others. They came to realize that being HIV-positive “doesn’t mean the end of your life.” For example:

Acceptance was difficult for me. So even taking medication daily was a problem. When I came here…they were teaching me that …if you are taking your medication, you can do things that people who are not sick do…Now, I am free to associate with anyone. I can make friends with anyone. I can go to school…I used to feel as though I am not part of those people.

(female, 18 years)

Sometimes even on our own you have a certain stigma. You feel embarrassed. You don’t feel free in your own life. But for me, [the program] has taught me a lot. I can even stand in public and talk about my status with confidence.

(female, 24 years)

By accepting their status, many youth were able to transcend their fears associated with living with HIV, including speaking with HCP about their status and picking up their ART medication from the clinic. Several described how they used to avoid asking HCP questions about living with HIV or sexual relationships out of concern that they would be judged—e.g., “Should I really ask? What will they think?” (male, 21 years). Project YES!, however, gave youth an opportunity to become familiar with and confident around the HCP, showing them that the HCP are resources for young people. As one explained:

Before we started having the meetings [for Project YES!], we were afraid of the counselors… But now we have learnt that the problems we have, they are the ones who can help us… They will teach you how to handle what you are facing… Now we look at them [the HCP] as family, where you are open and you can say anything to them.

(male, 19 years)

Several described how they had begun to collect their medication independently rather than sending someone else to collect their medication, since they no longer felt “afraid” that someone they know would see them there.

Project YES! also helped some youth develop “that confidence” and “muster up the courage” to disclose their HIV status to those people whom they could trust. A 20-year-old male participant explained that before the project, “I was scared to disclose my status—even to tell my best friend or some relatives of mine, for example, my younger brother or my sister. But being part of Project YES! helped me and I don’t feel ashamed of being HIV-positive.”

### “I am not alone”: Building a community for young people living with HIV

The final theme, discussed by about half of the youth, was that Project YES! helped them recognize that they are not the only ones living “a positive life.” Many youth felt isolated prior to the project and were “surprised” and “happy” to discover that many others had the same HIV status. A sense of community formed:

Project YES! has actually opened my eyes to see we are not alone. I am not alone. It’s made me to see that even if though I have a family at home, there is another family I have at Project YES! that would relate to everything I’m going through.

(male, 21 years)

Within this community, the youth were “free” to share their experiences and questions with others who understood their situations.

They depicted the individual and group meetings, together, as critical to helping them overcome their feelings of isolation. The individual sessions gave the youth an opportunity to open up to their YPM about any personal issues they were facing or ask questions about topics like condom use and sexual behavior, which they felt less comfortable raising in a group setting. These sessions were especially useful for youth who described themselves as being reserved. For example, “If you are a shy person, since you are just the two of you, you can ask about anything” (male, 17 years). They bonded with and were encouraged by their YPM. A 15-year-old male explained, “My peer mentor understands me, like, she knows me. She knows how to talk to me, how to cheer me up… She’s just family to me.” A few youth recommended identifying a dedicated private space for individual meetings with YPMs; some meetings had to take place in larger spaces where other people were coming in and out, given the busy nature of the clinic settings.

The group meetings complemented the individual meetings by fostering opportunities for learning and relationship-building with other youth living with HIV. One participant disliked that not everyone in his group chose to speak up during the group sessions. But most participants discussed how the group sessions allowed them to share their feelings, thoughts, questions, and challenges they are facing—i.e., the “things they may not even ask you at home” (female, 17 years). Many appreciated talking with others who could relate to their situations. Beyond the benefits of discussion and learning, several youth built lasting friendships with the others.

## Discussion

Our findings highlight the critical role of self-worth, identity, and sense of community for young people living with HIV to engage in self-care behaviors. Extant data supports this finding for other age groups. For example, among adults living with HIV in Zambia who disengaged from HIV care, three-quarters re-engaged after repeated clinic outreach [34]. Qualitative research has elucidated how positive social interactions and relationships with clinic providers can buffer against fears of living with HIV and stigma while making adults feel respected, thus supporting re-engagement in care [35, 36]. These feelings may have particular influence among adolescents and young adults during a distinct developmental stage characterized by identity formation and solidification. By addressing their concerns, fears, and questions, Project YES! helped free these youth from shame and fear and recognize their self-worth. They gained the courage to disclose their HIV status to important figures in their lives, which can have beneficial effects on social support [37]. They also became comfortable speaking openly with HCP and picking up their ART medication from the clinic—changes which were similarly observed in qualitative in-depth interviews with the HCP involved in delivering Project YES! [28, 30] and are increasingly recognized as critical in youth HIV care and treatment [38]. Together, these findings reinforce and clarify the Project YES! quantitative RCT results of reduced feelings of internalized stigma among intervention versus comparison participants [16].

Our findings strengthen and extend the limited evidence [15] for the use of peer relationships and modeling to improve youths’ HIV care and treatment outcomes. Project YES! participants attributed the intervention with increasing their motivation to take their HIV care “seriously” by regularly taking their medicine and attending clinic appointments. By receiving information, practical skills, encouragement from Project YES! peer mentors and other participants, they developed greater mindfulness towards their HIV care and enhanced future aspirations. Other projects emphasizing peer connection have found positive outcomes along the HIV care continuum in Zimbabwe [21] and Kenya [22]. Our findings support this evidence while underscoring Project YES’s distinctive approach of using peer mentors rather than peer educators who are actively engaged in role-modeling, listening, and problem-solving [16] and who receive extensive training and pay [19]. Findings lend credence to the intervention drawing on the five Cs for positive youth development [24]—particularly confidence and connection with peers and adults alike—and the Social Cognitive Theory constructs of self-efficacy, agency, and observational learning [25, 26]. Furthermore, these findings are consistent with the in-depth interviews conducted with YPM and HCP, both of whom highlighted YPMs’ unique abilities to connect with the youth given their similar age and shared experience of living with HIV [19, 28]. Interestingly, these findings were consistent for both youth who had viral load suppression and failure at midline data collection for the RCT. For those with viral load failure at midline, it may be that other factors outside of the scope of the project were at play in explaining the viral load failure, such as poverty, religious beliefs, or partner relationship dynamics [8].

The IDI highlighted another distinctive feature of the Project YES! intervention in its inclusion of peer-led individual and group counseling sessions, a combination which had great appeal among the youth interviewed. Group-level interventions for young people living with HIV have been praised for their ability to reach more adolescents using fewer resources than individual-level approaches [39]. Indeed, the youth interviewed described these sessions as essential to overcoming their feelings of isolation by interacting with peers who were also living with HIV. They no longer had to wonder if they were the only ones living a “positive life.” Our study, however, highlights the importance of also including individual-level sessions for youth—particularly those describing themselves as shy—to feel comfortable discussing sensitive topics. The individual sessions allowed the youth to bond with their YPM over shared challenges and experiences, which the YPM themselves highlighted as key to building a foundation of trust [19]. The combination of peer-led individual and group sessions thus supported complementary but distinct aims while appealing to a variety of needs among the youth. Existing projects for youth living with HIV have typically included either individual counseling [22] or group counseling [40, 41] but not both, and these sessions are often led by adults [40, 41]. We are aware of only one project which draws on a similar methodology integrating peer-led individual and group engagement with youth living with HIV [21], highlighting the importance of further exploring this approach as well as factors that influence its implementation in future studies. In addition to the combination of group and individual sessions, IDI underscored benefits of the Project YES! model in facilitating meetings between the youth and their regular HCP, which set a foundation on which these relationships could continue to grow beyond the life of the project. Additionally, the youth interviewed were supportive of the range of content covered in the sessions beyond ART adherence, such as nutrition/healthy living and sexual and reproductive health.

One topic which did not emerge as a theme concerned the involvement of caregivers in the Project YES! intervention. Some youth described benefits of having their caregivers included in the project, but nearly half did not have a caregiver attend the program. This is a limitation of the study given that caregiver involvement was not included as one of the sampling criteria. As a result, the findings on caregiver involvement did not reach saturation. The topic should be explored in future studies of Project YES! given that the family environment is recognized as playing a critical role in youths’ HIV care and treatment practices [8–10, 38]. Another study limitation concerns the potential for social desirability bias given the use of individual interviews, but we sought to account for this bias by drawing on methods to achieve qualitative rigor recommended by Tobin and Begley (described above) [32].

## Conclusions

Allowing youth to change their narrative from one of being “alone” and “scared” to having a family and confidence in their future, combined with an understanding of how ART works, can positively influence their ability to manage their HIV. One of the authors (JAD) had the honor of hearing Dr. Jonathan Mann, the first Director of the World Health Organization’s Global Programme on AIDS, speak in 1997 and he said something to the effect that before giving a youth a condom (to protect themselves from HIV) you need to give them a future. Project YES! was built on the premise that the greatest experts available to help AYA living with HIV achieve health outcomes and general wellness are young adults living with HIV. This study shows how addressing HIV is not only about medication; it is about self-worth, identity, and a sense of community. The program helped many accept their HIV status, develop greater feelings of self-worth, and reduce their fear and shame of living with HIV. Interacting with YPM and other peers living with HIV in both individual and group sessions helped the youth realize that they are not alone with their HIV status. Together, these findings reinforce the added value of using peer mentors in clinic settings to strengthen youths’ HIV care and treatment practices. They highlight that in order to make progress with addressing HIV among young people globally, we need to integrate personal and community agency into our programs and efforts.

## Supporting information

S1 File PLOS’ questionnaire on inclusivity in global research. (DOCX)
